# Ufl1 deficiency causes skin pigmentation by up-regulation of Endothelin-1

**DOI:** 10.3389/fcell.2022.961675

**Published:** 2022-09-02

**Authors:** Ke Wang, Hu-Ning Xu, Yi-Wen Wang, Jian Mao, Da Liu, Xiao-Jing Zhu, Yu-Sheng Cong, Miao Wang

**Affiliations:** ^1^ Key Laboratory of Aging and Cancer Biology of Zhejiang Province, School of Basic Medical Sciences, College of Life and Environmental Science, Hangzhou Normal University, Hangzhou, China; ^2^ The Second Affiliated Hospital, Hengyang Medical College, University of South China, Hengyang, China

**Keywords:** Ufmylation modification, Ufl1, Ufl1^f/f^ KRT14^Cre/+^, pigmentation, endothelin-1

## Abstract

Ufmylation (UFM1 modification) is a newly identified ubiquitin-like modification system involved in numerous cellular processes. However, the regulatory mechanisms and biological functions of this modification remain mostly unknown. We have recently reported that Ufmylation family genes have frequent somatic copy number alterations in human cancer including melanoma, suggesting involvement of Ufmylation in skin function and disease. UFL1 is the only known Ufmylation E3-like ligase. In this study, we generated the skin-specific *Ufl1* knockout mice and show that ablation of *Ufl1* caused epidermal thickening, pigmentation and shortened life span. RNA-Seq analysis indicated that *Ufl1* deletion resulted in upregulation of the genes involved in melanin biosynthesis. Mechanistically, we found that Endothelin-1 (ET-1) is a novel substrate of Ufmylation and this modification regulates ET-1 stability, and thereby deletion of *Ufl1* upregulates the expression and secretion of ET-1, which in turn results in up-regulation of genes in melanin biosynthesis and skin pigmentation. Our findings establish the role of *Ufl1* in skin pigmentation through Ufmylation modification of ET-1 and provide opportunities for therapeutic intervention of skin diseases.

## Introduction

Post-translational protein modification plays an important role in every aspect of cellular processes. Deficiency in protein modification is associated with numbers of human diseases (Themelis et al., 2005; Yang et al., 2016). Ubiquitination is the first and the one of the most prevalent post-translational protein modifications described so far. Besides Ubiquitination, several ubiquitin-like protein have been identified including SUMO, NEDD8, ISG15, Urm1, Atg8, Atg12, FAT10, and UFM1 (Hochstrasser, 2009). A growing number of studies have begun to unveil crucial revealed the role of NEDD8 (Zhou et al., 2016), ISG15 (Wolk et al., 2013; Malik et al., 2022), SUMO (Deyrieux et al., 2007; Zhou et al., 2018) in regulating skin development and diseases such as vitiligo, psoriasis, atopic dermatitis and inherited dermatological diseases. However, the biological function of the other ubiquitin-like proteins in the skin remains largely unknown.

UFM1 modification (Ufmylation) is a recently reported ubiquitin-like modification, which has one of each E1-like enzyme (Uba5), E2-like enzyme (Ufc1), E3-like ligase (Ufl1), and two UFM1 proteases UfSP1 and UfSP2 (Komatsu et al., 2004; Tatsumi et al., 2010). DDRGK1 (also known as UFM1-Binding Protein 1, UFBP1) is required for the maintenance of UFL1 ligase activity (Yoo et al., 2014). Dysfunction in the Ufmylation is implicated in numbers of human diseases (Azfer et al., 2006; Rubio et al., 2013; Hu et al., 2014; Yoo et al., 2014). E3-like ligase plays a key role in specific protein substrate recognition (Zheng & Shabek, 2017). Germline deletion of the *Ufl1* gene in mice led to severe anemia and cytopenia or even embryonic lethality (Zhang et al., 2015). Cardiomyocyte-restricted *Ufl1* knockout mice developed cardiomyopathy and heart failure (Li et al., 2018). Ablation of either *Ufl1* or *Ufbp1* led to significant loss of both Paneth and goblet cells and increased susceptibility to experimentally induced colitis (Cai et al., 2019). *Ufl1* deficiency causes kidney atrophy associated with disruption of endoplasmic reticulum homeostasis (Zhou Y. et al, 2021). These recent findings underscore the importance of the Ufl1 in development and physiology. Nonetheless, the function of Ufl1 in skin remains uncharacterized.

The skin is the largest organ in the body and the first line of defense against the outside world (Grice & Segre, 2011). In addition, external color affects animal survival and reproduction, as pigmentation can provide camouflage, protect against photodamage, affect body temperature, and facilitate social interaction (Weiner et al., 2007). Skin pigmentation results from the synthesis of melanin in pigment-producing cells, melanocytes, with subsequent distribution and transport of pigment granules to adjacent keratinocytes (KCs), which are involved in regulation of the proliferation and differentiation of melanocytes. Endothelin-1 (ET-1), encoded by *EDN1* gene, is a keratinocyte-derived factor that stimulate melanocytes located in the vicinity of keratinocytes by binding to endothelin receptors, which activates intracellular signaling cascades, and then regulates melanocyte proliferation and melanogenesis in the skin (Yada et al., 1991; Imokawa et al., 1992; Yohn et al., 1993; Hara et al., 1995). Moreover, it has been reported that addition of ET-1 to cultured human epidermal melanocytes results in increased expression of melanogenesis-related genes and tyrosinase activity (Imokawa et al., 1995). However, the underlying mechanism of ET-1 regulation in skin is poorly understood.

Our recent study shows that Ufmylation genes have frequent genomic alterations in tumors including melanoma (Zhou J. et al, 2021). To understand the role of Ufmylation in the skin, we generated skin *Ufl1* knockout (CKO) mice and found that *Ufl1* deletion results in epidermal thickening, pigmentation and shortened life span.

## Materials and methods

### Generation of *Ufl1* ^*f*/*f*^ KRT14^Cre/+^ mice and genotyping

Generation of a transgenic mouse line bearing an *Ufl1* conditional-knockout allele (*Ufl1*-floxed) described previously (Zhou Y. et al, 2021). To generate CKO mice, the *Ufl1*-floxed mice were crossed with KRT14^Cre/+^ mice. The primers used for PCR genotyping of the *Ufl1*-floxed mice and KRT14^Cre/+^ mice are described in Supplementary Table S1. All the mice used in this study were fed in Specific Pathogen Free (SPF) facilities. All animal experiments were performed according to the guidelines of the Animal Care and Use Committee of Hangzhou Normal University.

### Histology, immunohistochemistry and special staining

Morphologic changes in skin from both CTRL and CKO mice were determined by hematoxylin and eosin (HE) staining and Masson-Fontana staining. Skin tissue specimens were fixed overnight in 4% paraformaldehyde and embedded in paraffin. Three-micrometer-thick sections were generated and then immunohistochemically stained with antibodies against UFL1 (Bethyl Laboratories, A303-456A; dilution 1:100), Endothelin-1 (Proteintech, 12191-1-AP, dilution 1:100), or Ki-67 (Abcam, ab16667, dilution 1:200). The sections were scanned using an automated slide scanner to create high-resolution digital images (KFBIO). TUNEL staining was performed with a TUNEL detection kit (Recordbio Biological Technology) according to the manufacturer’s instructions. Images were acquired with an Eclipse C1 fluorescence microscope (NIKON).

### Cell culture and reagents

HaCaT cells were were purchased from Fenghbio (Hunan, China) and cultured in Keratinocytes-SFM (1×) (Gibco, 10725-018). Human embryonic kidney 293T (HEK293T) cells were purchased from the American Type Culture Collection and maintained in DMEM with 10% FBS (Procell, CM-0109) and 1% penicillin-streptomycin. The following three siRNAs against *Ufl1* was purchased from GenePharma; Ufl1-1: GGA​ACU​UGU​UAA​UAG​CGG​A; Ufl1-2: GAG​GAG​UAA​UUU​UUA​CGG​A; Ufl1-3: CUG​CUA​CCC​ACU​UCU​UUA​UTT. RNA interference was performed with Lipofectamine 3000 (Invitrogen) according to the manufacturer’s instructions. The sequence of shRNA targeting homo Ufl1 mRNA (NM_015323.5) is as follow: 5′-GCA​CGT​ATC​CGT​GGA​CTA​TTC-3’. Cycloheximide was purchased from Selleck; Toluidine Blue Staining Kit was purchased from Sangon Biotech.

### Elisa

ET-1 levels in serum and ear skin of mice were measured using an ELISA kit (mmbio, MM-0561M2, China) according to the manufacturer’s instructions.

### Western blot analysis

The antibodies used in this study were anti-UFL1 (Bethy Laboratories, A303-456A, dilution 1:1000), anti-UFL1 (Sigma, HPA030559, dilution 1:1000), anti-Endothelin-1 (Proteintech, 12191-1-AP, dilution 1:1000), anti-HA (Cell Signaling Technology, 3597S, dilution 1:1000), anti-Flag (Cell Signaling Technology, 3177S, dilution 1:1000), anti-UFM1 (Abcam, ab109305, dilution 1:1000) and anti-GAPDH (HuaAn Biotechnology, M1310-2, dilution 1:5000).

### RNA sequencing

Total RNA was extracted from ears of mice using Trizol reagent. The RNA integrity was assessed using the RNA Nano 6000 Assay Kit of the Bioanalyzer 2100 system (Agilent Technologies, CA, United States). Total RNA was used as input material for the RNA sample preparations. RNA-Seq libraries were prepared using the NEBNext^®^ Ultra RNA Library Prep Kit (Illumina, San Diego, CA) according to the manufacturer’s protocols. The RNA-seq libraries were sequenced using Illumina Novaseq platform and 150 bp paired-end reads were generated. FeatureCounts v1.5.0-p3 was used to count the reads numbers mapped to each gene. Differential expression analysis of twoconditions/groups (two biological replicates per condition) was performed using the DESeq2 R package (1.20.0). Genes with an adjusted *p*-value<0.05 found by DESeq2 were assigned as differentially expressed. RNA sequencing technology was provided by Novogene (Beijing, China).

### RNA extraction and real time-PCR

Total RNA was extracted with TRIzol reagent (Invitrogen, United States) according to the manufacturer’s instructions; cDNA was then prepared using 5×FastKing-RTsuperMix (TIANGEN). Quantitative real-time PCR (q-PCR) assays were performed using SYBR Green Supermix (Bio-Rad) with a CFX96 Real Time-PCR Detection System (Bio-Rad). The sequences of the primers used for q-PCR are described in Supplementary Table S2. Relative expression level of each transcript was normalized to murine beta-actin and GAPDH by using the 2 ^ (-delta delta Ct) method.

### Assays for ufmylation *in vivo* and *in vitro*

The *in vivo* Ufmylation assay has been described previously (Zhou Y. et al, 2021). Briefly, HEK293T cells were harvested after transfected with the appropriate constructs for 36 h. Cells were lysed by boiling in buffer (150 mM Tris-HCl (pH 8.0), 5% sodium dodecyl sulfate (SDS) and 30% glycerol) for 10 min. Cell lysates were diluted 20-fold with buffer A (50 mM Tris-HCl (pH 8.0), 150 mM NaCl, 0.5% NP-40 and 2 mM N-ethylmaleimide) and protease inhibitor cocktails (Roche). After incubation with anti-Flag M2 Affinity Gel (Sigma) overnight at 4°C, the immunoprecipitates were subjected to SDS polyacrylamide gel electrophoresis (SDS-PAGE) followed by western blot analysis.

GST-tagged ET-1 were ectopically expressed in BL21 (DE3) cells and purified using Glutathione Sepharose (GE Healthcare). *In vitro* Ufmylation assay was performed as described previously (Liu et al., 2020).

### Statistics and reproducibility

Western blot images were acquired with an MP 5000 VersaDoc imaging system (BioRad). GraphPad Prism 8 was used for all statistical analyses (GraphPad Software). Student’s *t*-test was performed for all quantitative data between different groups, and the statistical significance was labeled as *p < 0.05, **p < 0.01, ***p < 0.001, n.s. no significant. Each experiment was repeated independently with similar results.

## Result

### Generation of Ufl1^f/f^ KRT14^Cre/+^mice

**FIGURE 1:**
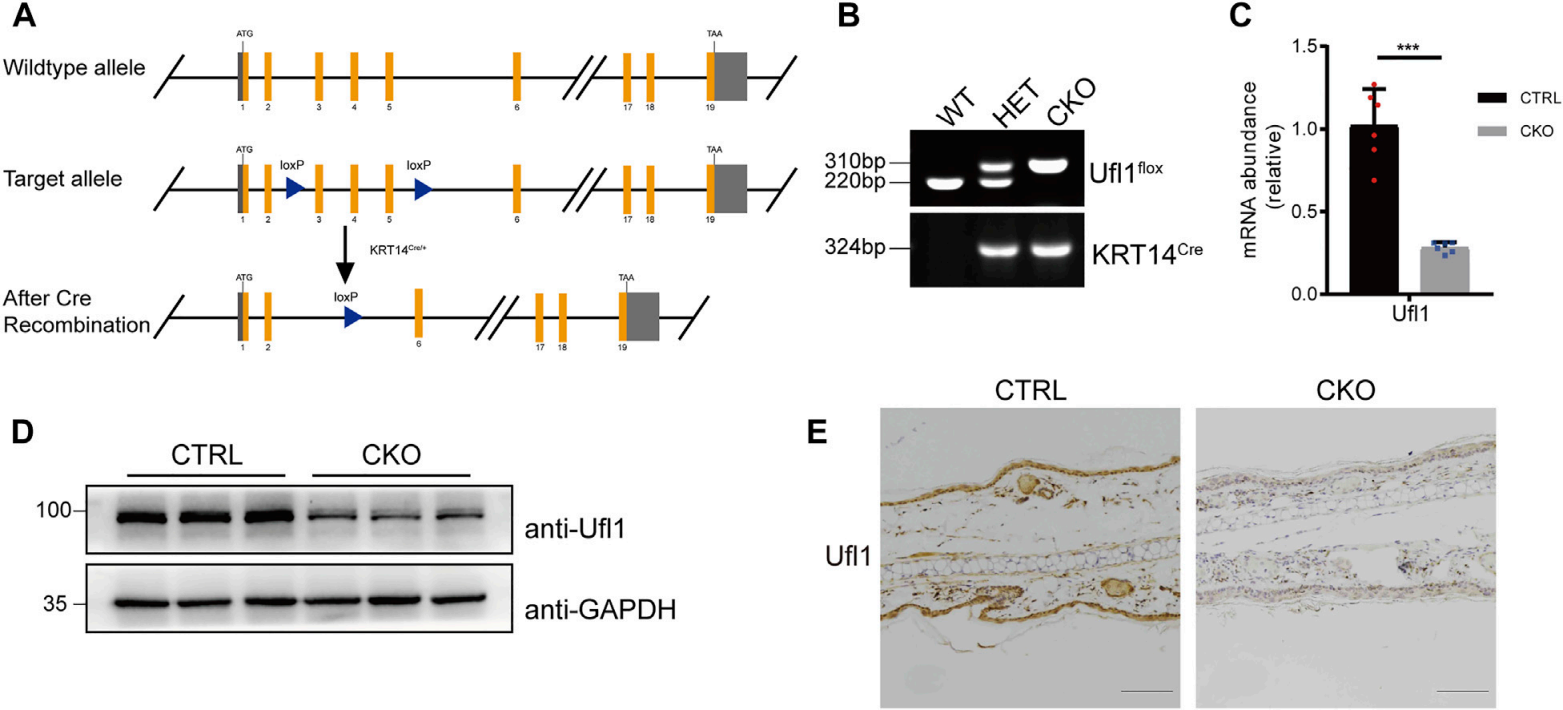
Generation of *Ufl1* ^*f*/*f*^ KRT14^Cre/+^ mice. **(A)** Targeting strategy to generate *Ufl1* conditional knockout mice. *Ufl1* ^*f*/*f*^ mice were crossed with KRT14^Cre/+^ transgenic mice to obtain conditional skin knockout mice. **(B)** PCR-based genotyping of wild-type and floxed alleles of Ufl1 and KRT14-Cre allele. **(C)** q-PCR analysis of *Ufl1* gene expression in the skin of 3-month-old CTRL and CKO mice (*n* = 6). **(D)** Ufl1 expression was detected by Western blot in the skin of 3-month-old CTRL (*Ufl1* ^f/+^, *Ufl1* ^*f/f*^ or *Ufl1* ^*f/+*^ KRT14^Cre/+^), CKO (*Ufl1* ^*f/f*^ KRT14^Cre/+^) mice. **(E)** Immunohistochemical staining (IHC) of *Ufl1* proteins in sections of ear skin from CTRL and CKO. ***p < 0.001, unpaired Student’s test. Error bars represent ± s.d. The scale bar represents 100 μm.

We have previously generated mice carrying *Ufl1* flox alleles (Zhou Y. et al, 2021), which were crossed with keratin 14 promoter-driven Cre transgenic (KRT14^Cre/+^) mice to achieve skin knockout of *Ufl1* (*Ufl1* ^*f*/*f*^ KRT14^Cre/+^, hereafter referred to as CKO mice) (Figure 1A). Genomic insertion of the loxP sites in the *Ufl1* gene and KRT14^Cre/+^ were confirmed by PCR (Figure 1B), and the expression of *Ufl1* in the skin of CKO mice and CTRL mice (*Ufl1* ^*f*/+^or *Ufl1* ^*f*/*f*^, hereafter referred to as CTRL mice) were analyzed by q-PCR, western blotting, and immunohistochemical staining. These analyses demonstrated that *Ufl1* was specifically deleted in the skin (Figures 1C–E), while it was expressed normally in other tissues (Supplementary Figure S1A). As expected, the global levels of UFM1-conjugated (Ufmylated) proteins were reduced in skin of CKO mice (Supplementary Figure S1B). These data indicated that skin-specific *Ufl1*-knockout mice were successfully generated.

### Loss of Ufl1 causes severe skin abnormalities in mice

**FIGURE 2:**
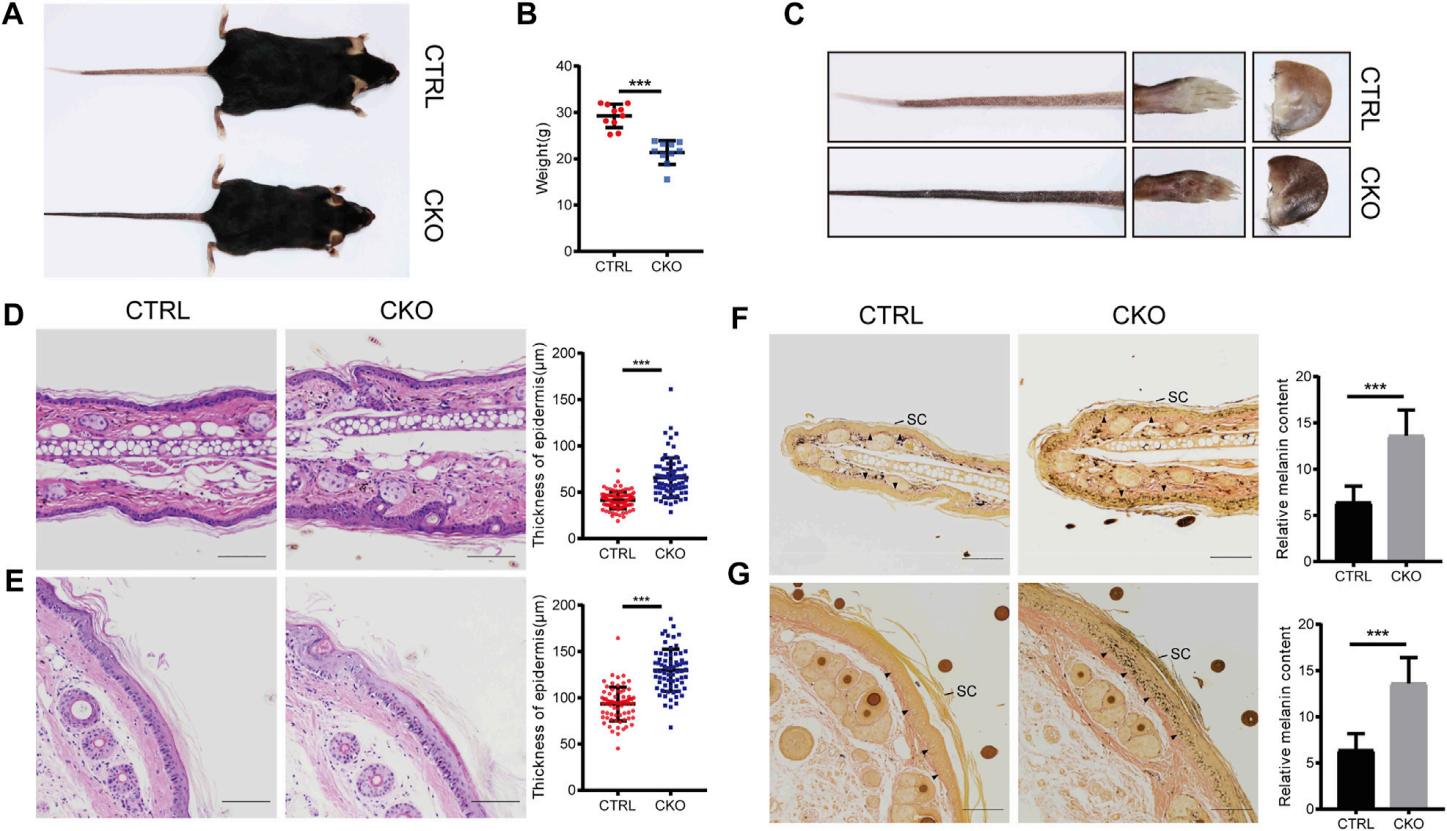
Loss of Ufl1 causes severe skin abnormalities in mice. **(A)** Images of mice with the indicated genotypes. **(B)** Body weight of mice in A. **(C)** Paws, tails and ears of the indicated mice as shown in A. **(D,E)** Tails and ears sections from CTRL and CKO mice were stained with hematoxylin and eosin (H&E) and epidermal thickness. **(F,G)** Masson-Fontana staining of the tail and ear sections of mice in C, and melanin quantification of the indicated tail and ear samples. Arrowheads mark the dermal-epidermal border in panels. SC, stratum corneum. ***p < 0.001, unpaired Student’s test. Error bars represent ± s.d. The scale bar represents 100 μm.

The CKO mice were born at a Mendelian ratio without apparent morphologically different compared with the CTRL mice. The analysis of skin permeability with toluidine blue staining revealed no difference between CTRL and CKO mice in neonates (birth day 0) (Supplementary Figure S2A). Notably, at 3 months of age, CKO mice showed a significantly reduced body weight and exhibited enhanced skin pigmentation at sites with little or no hair, such as the ears, tails and footspads (Figures 2A–C). Compared to CTRL mice, CKO mice had shortened lifespan (Supplementary Figure S2B), reduced body weight and showed a hunchback with flflaky skin, sparse hair and pigmentation at 16 months of age (Supplementary Figures S2C–E). Moreover, CKO mice exhibited increased epidermis thickness as assayed by HE staining in the ear and tail at 3 months old (Figures 2D,E). To examine whether absence of epidermal *Ufl1* results in defective keratinocyte proliferation or apoptosis, we analyzed the epidermis of ears in 3 months mice. Results indicate that the absence of *Ufl1* did not affect apoptosis of keratinocytes (KCs) in the epidermis as revealed by TUNEL staining, while the proliferation of keratinocytes was increased in the CKO mice, especially in the basal layer and hair follicle as assessed by Ki67 staining (Supplementary Figure S2F).

The color change observed in skin of CKO mice by Masson-Fontana staining suggests that *Ufl1* may involve in melanogenesis. Consistent with the darkened coloration, we observed that melanin, which was normally located at dermis in CTRL mice, appeared at both the epidermis and dermis in CKO mice, specifically in hairless regions (Figures 2F,G). In addition, we observed that most of melanin was located in the basal layer in CKO mice, but it was also detected in suprabasal keratinocytes, and the melanin-positive cells extended into the stratum corneum, where they accumulated as dead, pigmented debris (the end product of keratinocyte differentiation). Whereas, no change in the color was observed in the dermis of CKO mice. These data suggest that Ufl1 plays a critical role in melanogenesis.

### Global transcriptome analysis of ear

**FIGURE 3:**
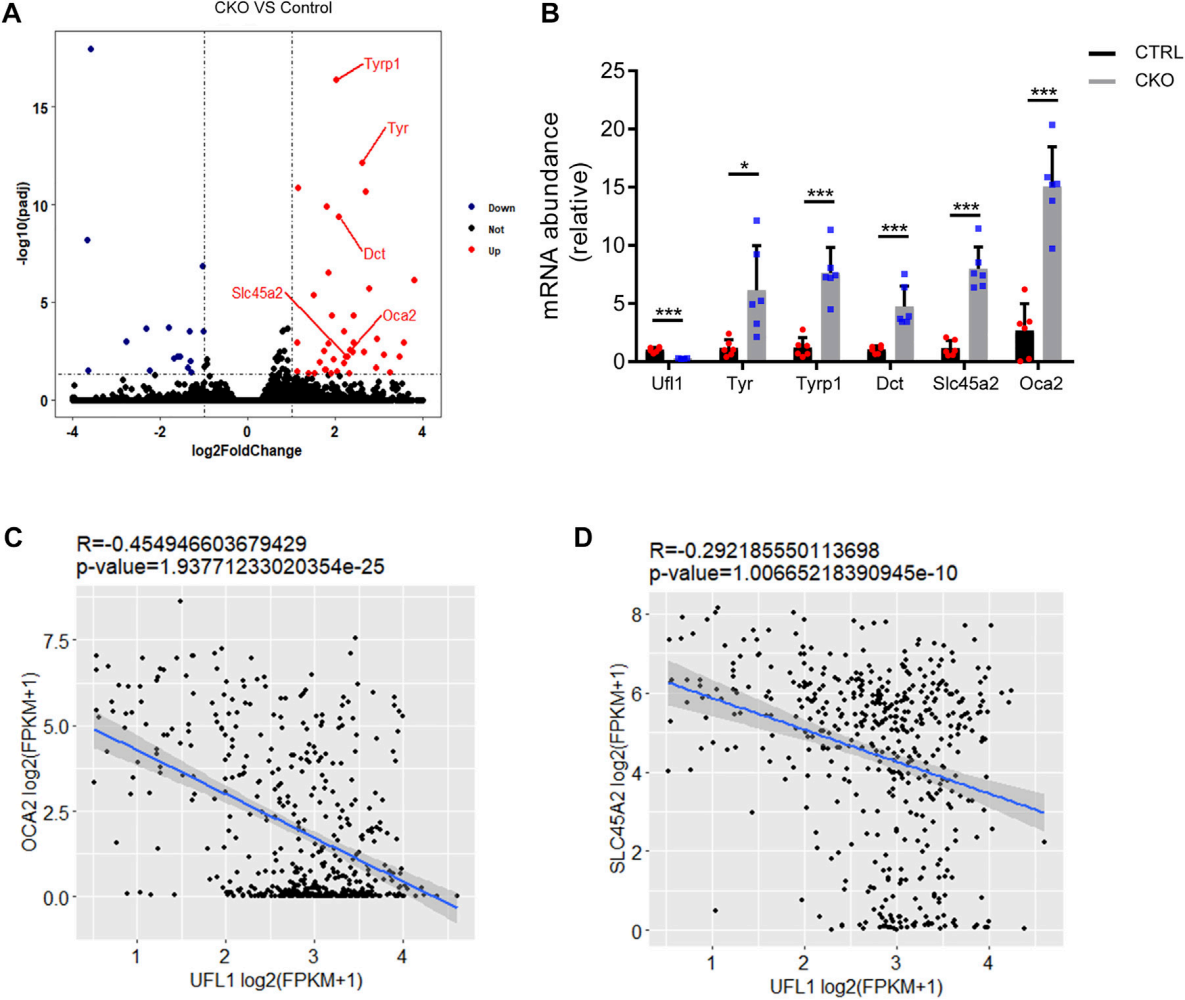
Global transcriptome analysis of ear. **(A)** Volcano plot of downregulated (blue) and upregulated (red) transcripts in CKO mice skin. Genes in melanin biosynthesis differentially expressed are marked by red lines. **(B)** q-PCR confirmation of mRNA expression of melanin biosynthesis-related genes. **(C,D)** Pearson’s correlation between *Ufl1* and OCA2 or SLC45A2 in The Cancer Genome Atlas (TCGA) SKCM. Plots show the Spearman’s correlation and OCA2 or SLC45A2 with UFL1 mRNA level from RNA-seq data in TCGA melanoma calculated. R and *p* values are shown. *p < 0.05, ***p < 0.001, unpaired Student’s test. Error bars represent ± s.d.

To understand the characteristic features of pigmentation in CKO mice, we performed RNA sequencing analysis (RNA-seq) in the CTRL and CKO ears at 3 months of age and the results indicated that the expression of genes belonging to enzymatic components of melanin biosynthesis including tyrosinase (Tyr), tyrosinase-related protein 1 (Tyrp1), melanocyte-Specific Transporter Protein (Oca2), dopachrome tautomerase (Dct) and Solute Carrier Family 45 Member 2 (Slc45a2), was markedly upregulated in ears of CKO mice (Figure 3A). These results were further confirmed by q-PCR analysis (Figure 3B). We also examined the expression of other genes involved in melanin synthesis, such as Mlana, Pmel and Trpm1, which are considered as biomarkers for melanoma (Wang et al., 2011; Brozyna et al., 2017; Zhang et al., 2021) and the results showed that these genes were significantly upregulated as well in CKO mice (Supplementary Figure S3). Furthermore, by using RNA-seq data from The Cancer Genome Atlas (TCGA) melanoma cohort (Tang et al., 2017), we found that the mRNA level of UFL1 was negatively correlated with those of well-known targets downstream from melanin biosynthesis pathway, including OCA2 and SLC45A2 (Figures 3C,D). Taken together, these results suggest that *Ufl1* plays a vital role in melanin biosynthesis *in vivo*.

### Ufl1 deletion promotes the secretion of Endothelin-1

**FIGURE 4:**
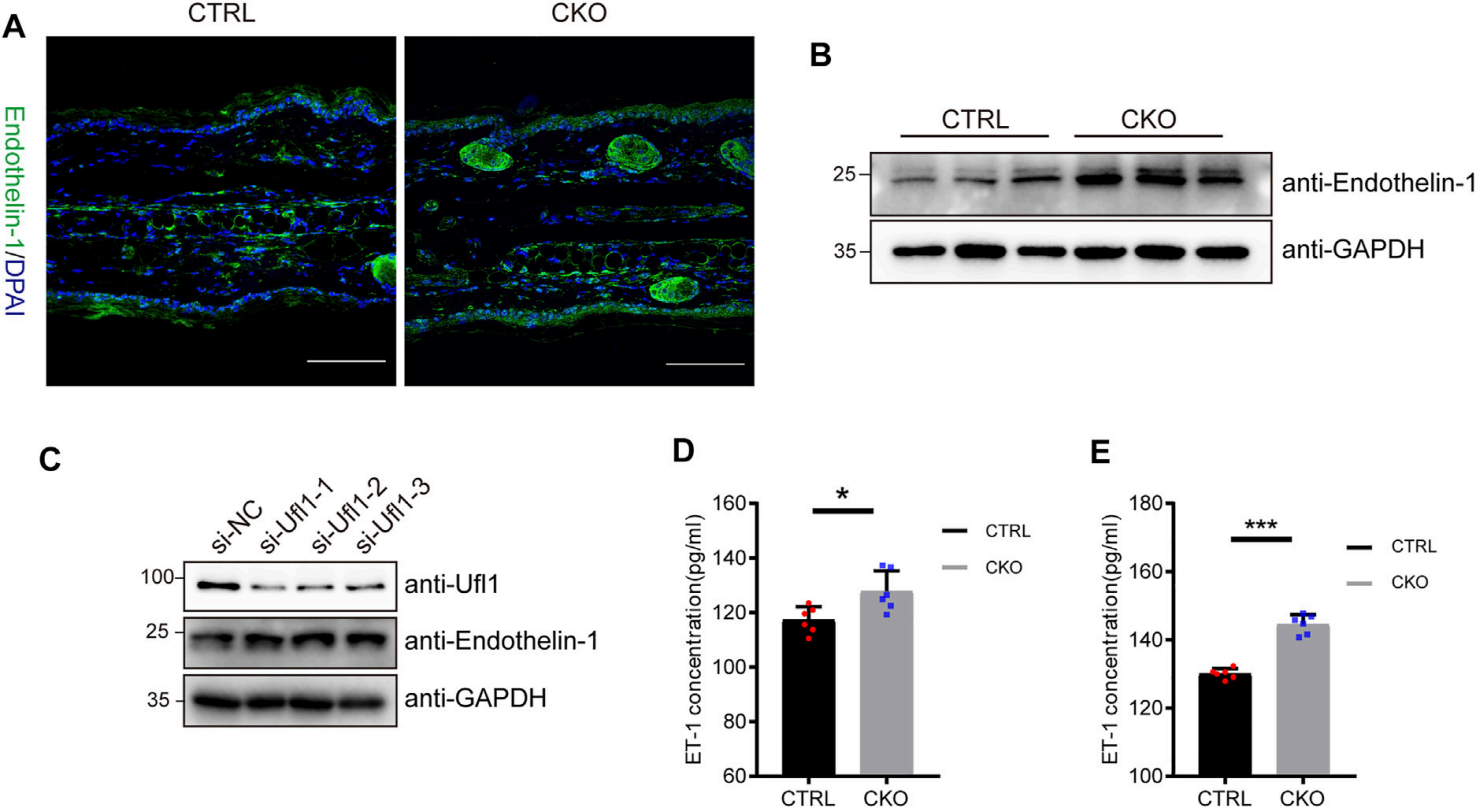
Ufl1 deficiency promote the expression and secretion of Endothelin-1 (ET-1). **(A)** Immunofluorescence staining for mice ears sections. The images showed ET-1 staining (green), and DAPI staining (blue) as a nuclear counterstain (upper panel). **(B)** Western blot analysis of ET-1 expression in the ears of 3-month-old CRTL and CKO mice (*n* = 3). **(C)** Western blot analysis of ET-1 expression in the HaCaT cells with *Ufl1* depletion. si-RNA, small interfering RNA; NC, negative control. **(D,E)** ET-1secretion in the plasma and ears was analyzed by ELISA (*n* = 6). *p < 0.05, ***p < 0.001, unpaired Student’s test. Error bars represent ± s.d. The scale bar represents 100 μm.

Melanocytes in the epidermis contribute to human skin pigmentation by synthesizing the melanin, thereby neighboring keratinocytes receive and distribute it to upper layers of the skin. Endothelin-1 (ET-1) is a keratinocyte-derived factor that is involved in regulating the proliferation of melanoblasts and melanocytes, as well as the differentiation of melanocytes. To assess whether ET-1 is differentially expressed in the CTRL and CKO mice, we performed immunofluorescent staining with anti ET-1 antibody. As shown in Figure 4A, ET-1 fluorescence intensity was markedly increased in the epidermis of CKO mice. Consistently, we found that the protein levels of ET-1 dramatically upregulated in the CKO mice without significant changes in *ET-1* messenger RNA levels (Figure 4B and Supplementary Figure S4A). Similar results were observed in Ufl1-depleted HaCaT cells (Figure 4C and Supplementary Figure S4B). Conversely, overexpression of Ufl1 in HaCaT keratinocyte cells resulted in down-regulated expression of ET-1 (Supplementary Figure S4C). We then evaluated secretion of ET-1 in mice plasma and ears by ELISA and found that the level of secreted ET-1 was upregulated in CKO mice (Figures 4D,E). These results demonstrate that the loss of Ufl1 led to upregulated ET-1 expression and secretion.

### Ufl1 deletion upregulates Endothelin-1 synthesis

**FIGURE 5:**
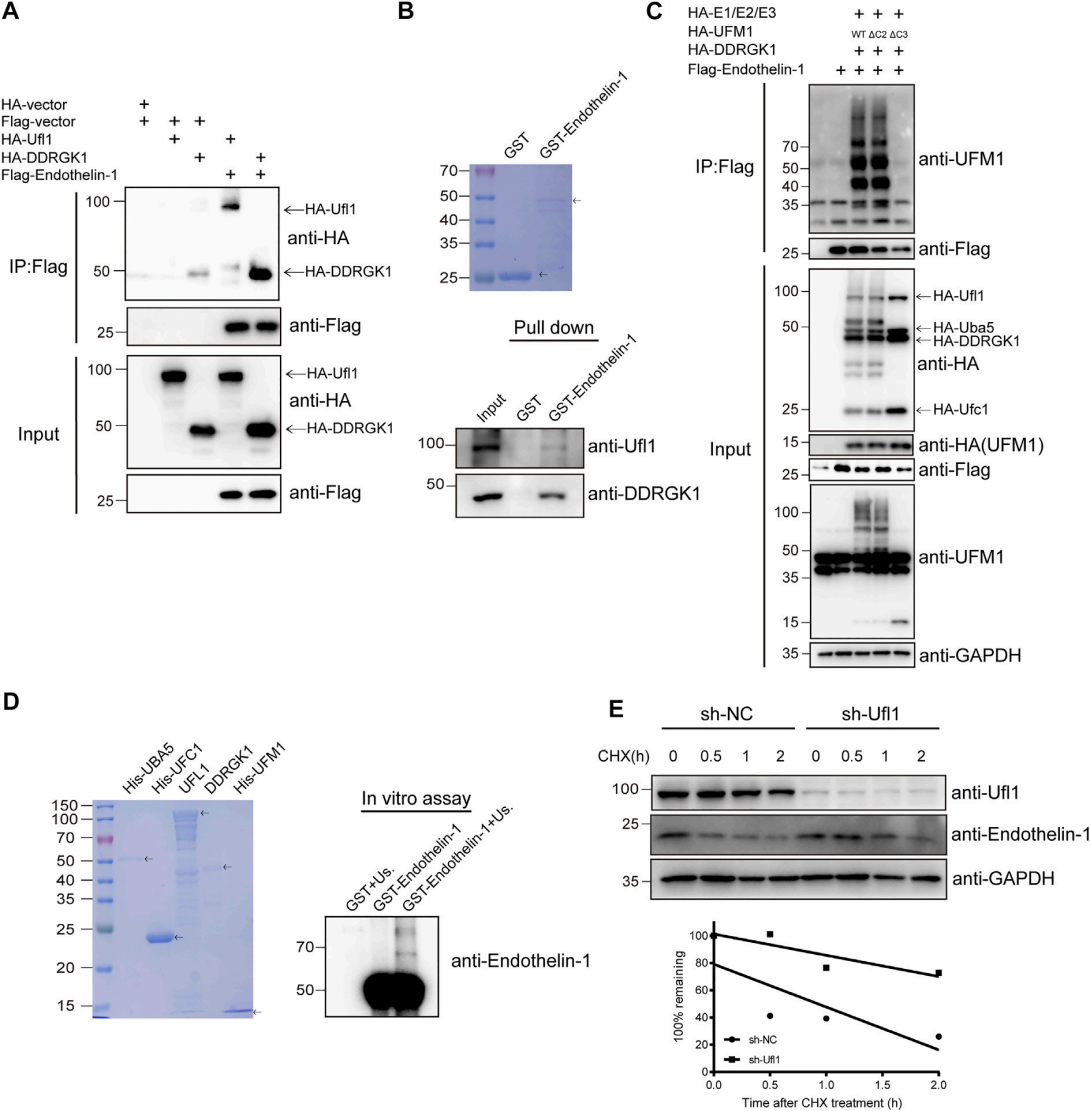
Ufl1 deletion affects Endothelin-1 synthesis. **(A)** Western blot analysis of the mutual interactions between Ufl1, DDRGK1 and ET-1 in HEK293T cells by co-immunoprecipitation. **(B)** *In vitro* binding assays between ET-1 and Ufl1 or DDRGK1. **(C)** Ufmylation of ET-1 was analyzed by western blot with anti-UFM1 antibody in HEK293T cells expressing the Ufmylation system components. **(D)** *In vitro* Ufmylation assay of ET-1. Us., UFMylation system components (consisting of UBA5, UFC1, UFL1, DDRGK1 and UFM1). **(E)** ET-1 stability was examined by western blot in UFL1-depletion HaCaT cells (sh-UFL1) compared to control HaCaT cells (sh-NC). The cells were treated with 100 µg ml−1 cycloheximide (CHX) for the indicated times. The graph represents quantification of the ET-1 protein levels.

To understand how deletion of Ufl1 resulted in upregulation of ET-1, we assessed whether ET-1 is a substrate of Ufmylation modification, given that Ufl1 is the only known E3 ligase of Ufmylation. Immunoprecipitation analysis indicated that UFL1, DDRGK1 and ET-1 were capable of binding to each other (Figure 5A and Supplementary Figure S4D). Furthermore, the *in vitro* pull-down assay with purified recombinant proteins (GST-ET-1, UFL1 and DDRGK1) showed that ET-1 directly interacted with UFL1 and DDRGK1 (Figure 5B). To determine whether ET-1 can be Ufmylated, we co-expressed ET-1 in HEK293T cells with the Ufmylation components UBA5, UFC1, UFL1, UFM1 and DDRGK1. The Ufmylation assays showed that wild-type UFM1 (UFM1WT) and an active form of UFM1 with an exposed carboxy (C)-terminal glycine 83 residues (UFM1∆C2) could conjugate to ET-1, but not an inactive form of UFM1 lacking the C-terminal glycine 83 residues (UFM1∆C3) (Figure 5C). In addition, the *in vitro* Ufmylation assay further demonstrated that ET-1 is a substrate of Ufmylation modification (Figure 5D). Moreover, we examined the effect of Ufmylation on ET-1 stability by cycloheximide chase assay and found that ET-1 stability was increased after depletion of UFL1 (Figure 5E). Thus, these data suggest that Ufl1 regulates ET-1 stability through Ufl1-mediated Ufmylation modification.

## Discussion

In the present study, we showed that ablation of Ufl1 caused epidermal thickening, pigmentation and reduced life-span of animals. Our RNA-seq analysis indicated that the expression of genes of enzymatic components of melanin biosynthesis were significantly upregulated in CKO mice. We further identified ET-1 as a novel substrate of Ufmylation and showed that Ufl1 is involved in skin pigmentation by regulating ET-1 expression and secretion through Ufmylation modification.

Among the constituent cells present in the skin, KCs secrete a mass of mitogenic or melanogenesis factors that are recognized by their corresponding receptors on melanocytes. These KCs-derived cytokines or growth factors play essential roles in proliferation, differentiation and melanogenesis (Slominski et al., 2004). ET-1 releases from keratinocytes and binds to EDNRs on melanocytes, thereby transmitting signaling cascade to participate in the melanogenesis process. The hyperpigmentation observed in basal cell carcinoma (BCC) and seborrheic keratosis (SK) is associated with enhanced expression of keratinocyte-derived ET-1 (Teraki et al., 1996; Lan et al., 2005). Consistently, we found that pigmentation in CKO mice is associated with up-regulation of ET-1. It is known that increased ET-1 not only can cause hyperpigmentation, but also associated with atopic dermatitis (Aktar et al., 2015; Nakahara et al., 2018) and psoriasis (Bonifati et al., 1998), the well-known chronic pruritic dermatosis. Serum ET-1 levels have been reported to be elevated in patients with atopic dermatitis and psoriasis and correlate with disease severity. Interestingly, our RNA-seq analysis indicates that inflammation-related signaling pathways were upregulated in CKO mice, suggesting that Ufl1-deficient mice also have an inflammatory response (Supplementary Figure S5).

It is worth to mention that the KRT14-Cre is also expressed in dental epithelium and this may affect dental development. Indeed, we found that CKO mice exhibited tooth morphological abnormalities (including breaking, twisting, and lengthening). Thus, it is possible that reduced body weight observed in CKO mice may be due to their feeding difficulty because of dental abnormalities.

In summary, our study revealed the critical role of Ufl1 in skin pigmentation, which may provide insights for a better understanding of the mechanisms of pigmentary disorders and for therapeutic strategies.

## Data Availability

The datasets presented in this study can be found in online repositories. The names of the repository/repositories and accession number(s) can be found in the article/Supplementary Material.
