# Supplementary material for: Ufl1 deficiency causes skin pigmentation by up-regulation of Endothelin-1
Source: Front Cell Dev Biol. 2022 Sep 2;10:961675. doi: 10.3389/fcell.2022.961675 (PMC9478483; doi:10.3389/fcell.2022.961675)
Supplement: Supplementary file 1 [file Table1.pdf]

Supplementary Material

1 Supplementary Tables

Table S1. The primers used for genotyping.

Genotyping Primers	Sequence	Species
1-1 *Ufl1*-tF1	AGCAGACTGAGCAGTTTGACTGAA	Mouse
1-2 *Ufl1*-tR1	AAAATGACCAGAGGAAATGCCTG	Mouse
2-1 *Ufl1*-5tF1	CCACACTTCATGACCTAGGGGTGA	Mouse
2-2 Common-En2R	CCAACTGACCTTGGGCAAGAACAT	Mouse
3-1 *Zmk*-2F4	CATCGCATTGTCTGAGTAGGTG	Mouse
3-2 *Ufl1*-3tR1	AGCCCAATCCAAGGCAATTTCTCT	Mouse
4-1 oIMR3069	TTCCTCAGGAGTGTCTTCGC	Mouse
4-2 oIMR3070	GTCCATGTCCTTCCTGAAGC	Mouse
5-1 oIMR7338	CTAGGCCACAGAATTGAAAGATCT	Mouse
5-2 oIMR7339	GTAGGTGGAAATTCTAGCATCATCC	Mouse

Table S2. The primers used for q-PCR.

Q-PCR Primers	Sequence	Species
Ufl1- Forward	CTGGGACAACTGATTGATGAGAA	Mouse
Ufl1- Reverse	AGGAAGGTCATAGGCTTTACACA	Mouse
Tyr- Forward	CTCTGGGCTTAGCAGTAGGC	Mouse
Tyr- Reverse	GCAAGCTGTGGTAGTCGTCT	Mouse
Tyrp1- Forward	CCCCTAGCCTATATCTCCCTTTT	Mouse
Tyrp1- Reverse	TACCATCGTGGGGATAATGGC	Mouse
Dct- Forward	GTCCTCCACTCTTTTACAGACG	Mouse
Dct- Reverse	ATTCGGTTGTGACCAATGGGT	Mouse
Oca2- Forward	ATGCGCCTAGAGAACAAAGAC	Mouse
Oca2- Reverse	TAGCAGGTTTGACGGTCAGC	Mouse
Slc45a2- Forward	CCGACTGACACCCATACCTAT	Mouse
Slc45a2- Reverse	ATGCTGTGCATGACAAGTCTC	Mouse
Mlana- Forward	AGACGCTCCTATGTCACTGCT	Mouse
Mlnan- Reverse	TCAAGGTTCTGTATCCACTTCGT	Mouse
Pmel- Forward	GAGCTTCCTTCCCGTGCTT	Mouse
Pmel- Reverse	TGCCTGTTCCAGGTTTTAGTTAC	Mouse
Trpm1-Forward	AGGTGGTCCTAACGTGGTTTC	Mouse
Trpm1-Reverse	TCCTTCGTCGCAGTATTTGTG	Mouse

Edn1- Forward	GCACCGGAGCTGAGAATGG	Mouse
Edn1- Reverse	GTGGCAGAAGTAGACACACTC	Mouse
β-actin- Forward	GACATGGAGAAGATCTGGCA	Mouse
β-actin Reverse	GGTCTCAAACATGATCTGGGT	Mouse
EDN1- Forward	AGAGTGTGTCTACTTCTGCCA	Human
EDN1- Reverse	CTTCCAAGTCCATACGGAACAA	Human
GAPDH- Forward	TGCTAAGCAGTTGGTGGTGCAGGA	Human
GAPDH- Reverse	CGGAGTCAACGGATTTGGTCGTAT	Human
