# Supplementary material for: Ufl1 deficiency causes skin pigmentation by up-regulation of Endothelin-1
Source: Front Cell Dev Biol. 2022 Sep 2;10:961675. doi: 10.3389/fcell.2022.961675 (PMC9478483; doi:10.3389/fcell.2022.961675)
Supplement: Supplementary file 2 [file Image1.pdf]

Supplementary Date

1 Supplementary Figures

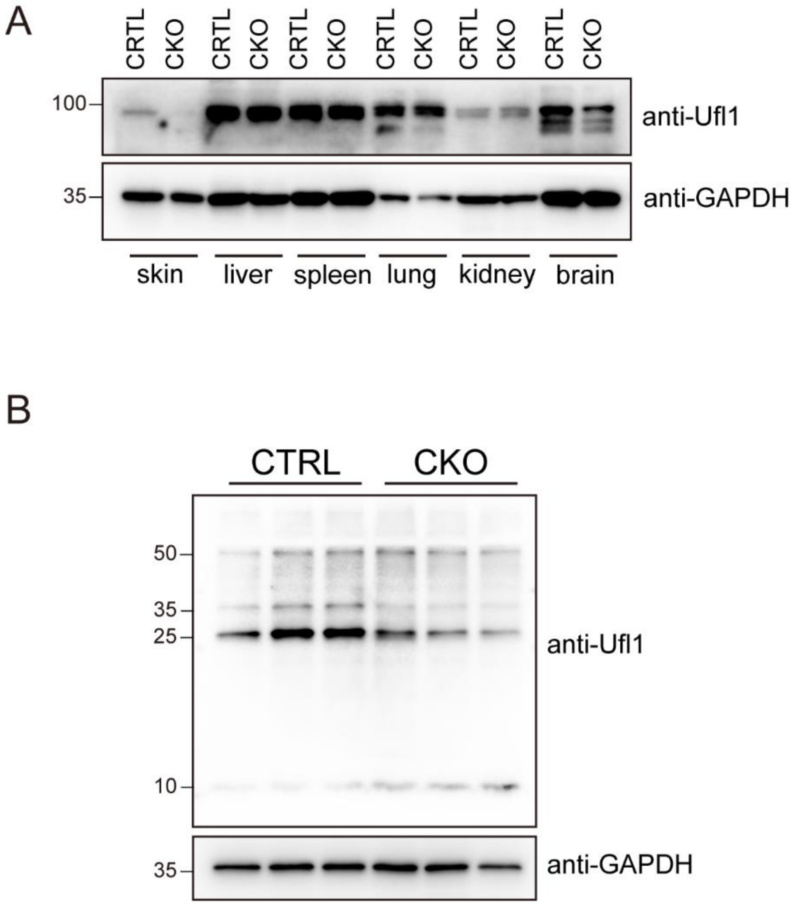

Supplemental Figure 1. (A) Western blot analysis of *Ufl1* expression in the skin, liver, spleen, lung, kidneys and brain of 3-month-old CTRL and CKO mice. **(B)** Representative Western blot of UFM1-conjugated proteins in CTLR and CKO mice (n=3).

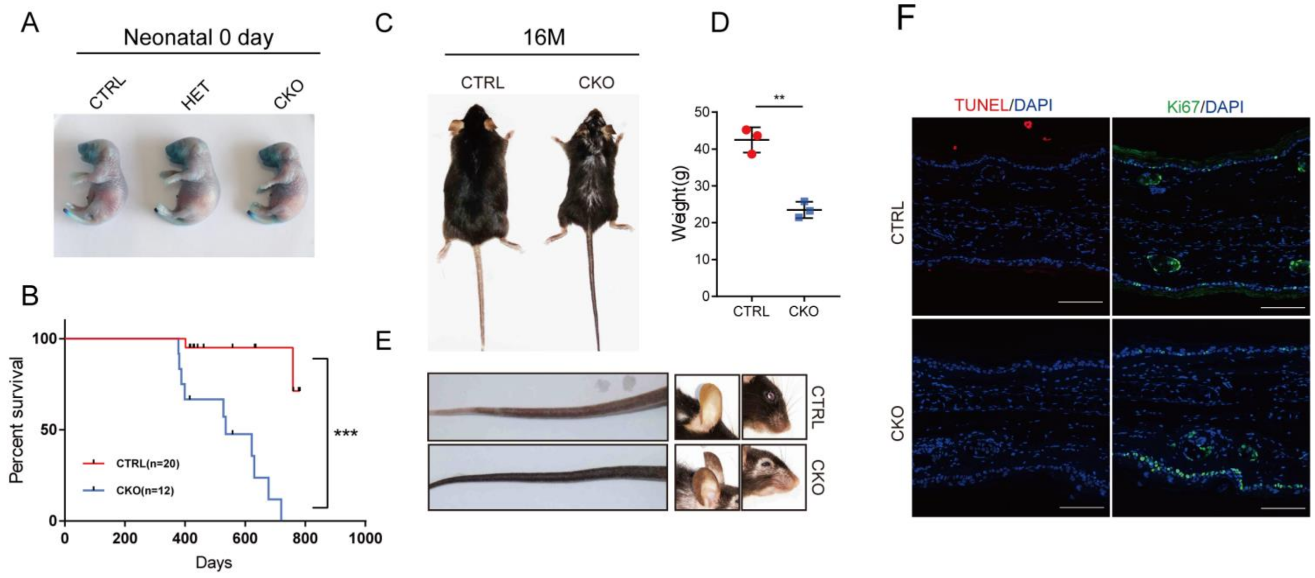

Supplemental Figure 2. (A) CTRL, HET (*Ufl1*$^{f/+}$ KRT14$^{Cre/+}$) and CKO neonates (birth day 0) were stained with toluidine blue. **(B)** Kaplan–Meier plot showing Ufl1-free survival of the indicated mice. **(C)** Images of mice with the indicated genotypes in 16-month-old mice. **(D)** Body weight of mice in C (n=3). **(E)** Tails and ears of the indicated mice as shown in C. **(F)** Immunofluorescence images of ear sections stained with DAPI (blue), TUNEL (red) and Ki67 (green)respectively. ***p＜0.001, unpaired Student's test. Error bars represent ± s.d. The scale bar represents 100 μm.

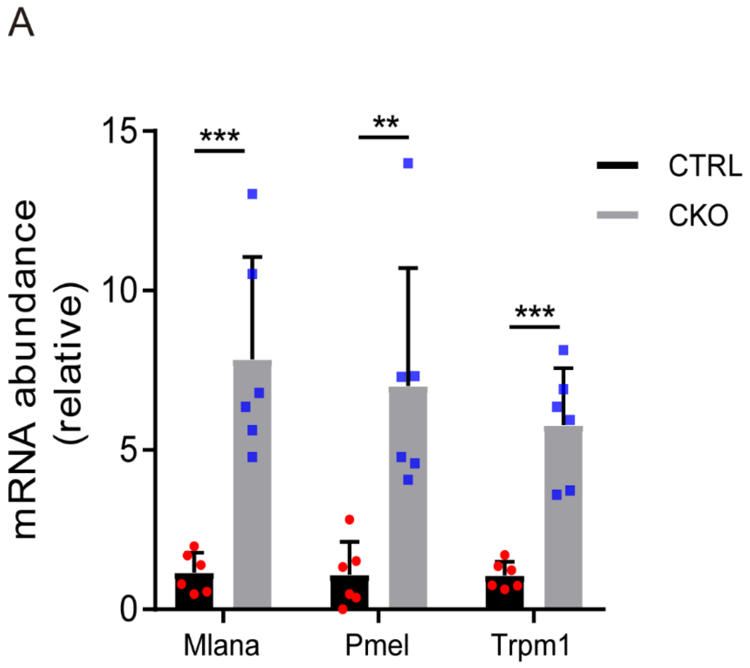

Supplemental Figure 3. Expression levels of other melanogenesis-related genes, assessed by q-PCR (n=6). **p＜0.01, ***p＜0.001, unpaired Student's test. Error bars represent ± s.d.

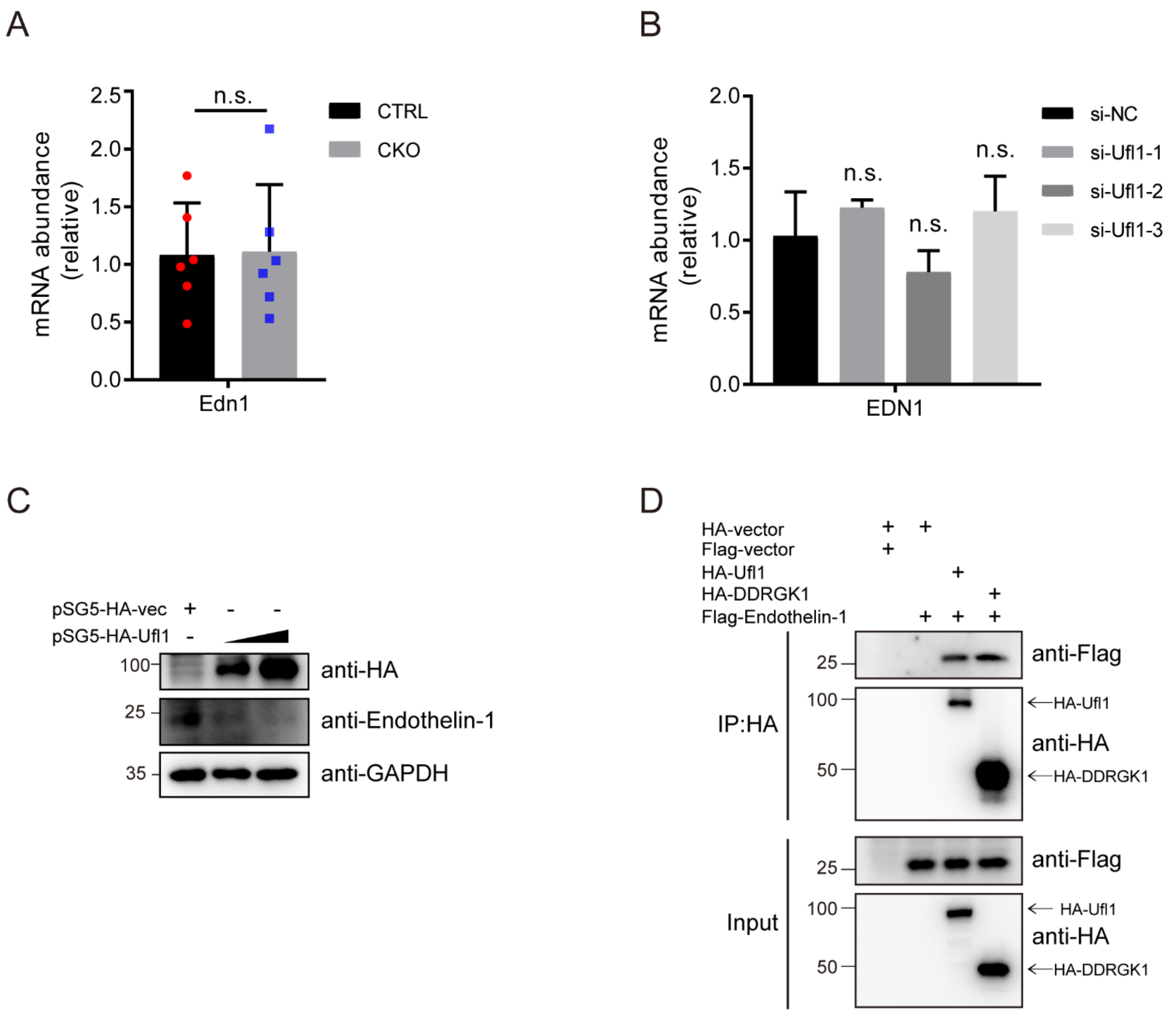

Supplemental Figure 4. (A) q-PCR analysis of the relative mRNA expression of Edn1 in CTRL mice and CKO mice (n=6). **(B)** q-PCR analysis of the relative mRNA expression of EDN1 in HaCaT cells with UFL1 depletion. **(C)** HaCaT cells were transfected with either pSG5-HA-plasmid, HA-*Ufl1* plasmid. ET-1 and HA-Ufl1 protein expression were analyzed by western blot. **(D)** Western blot analysis of the mutual interactions between exogenous ET-1, Ufl1 and DDRGK1 in HEK293T cells by co-immunoprecipitation. n.s. no significant.

A

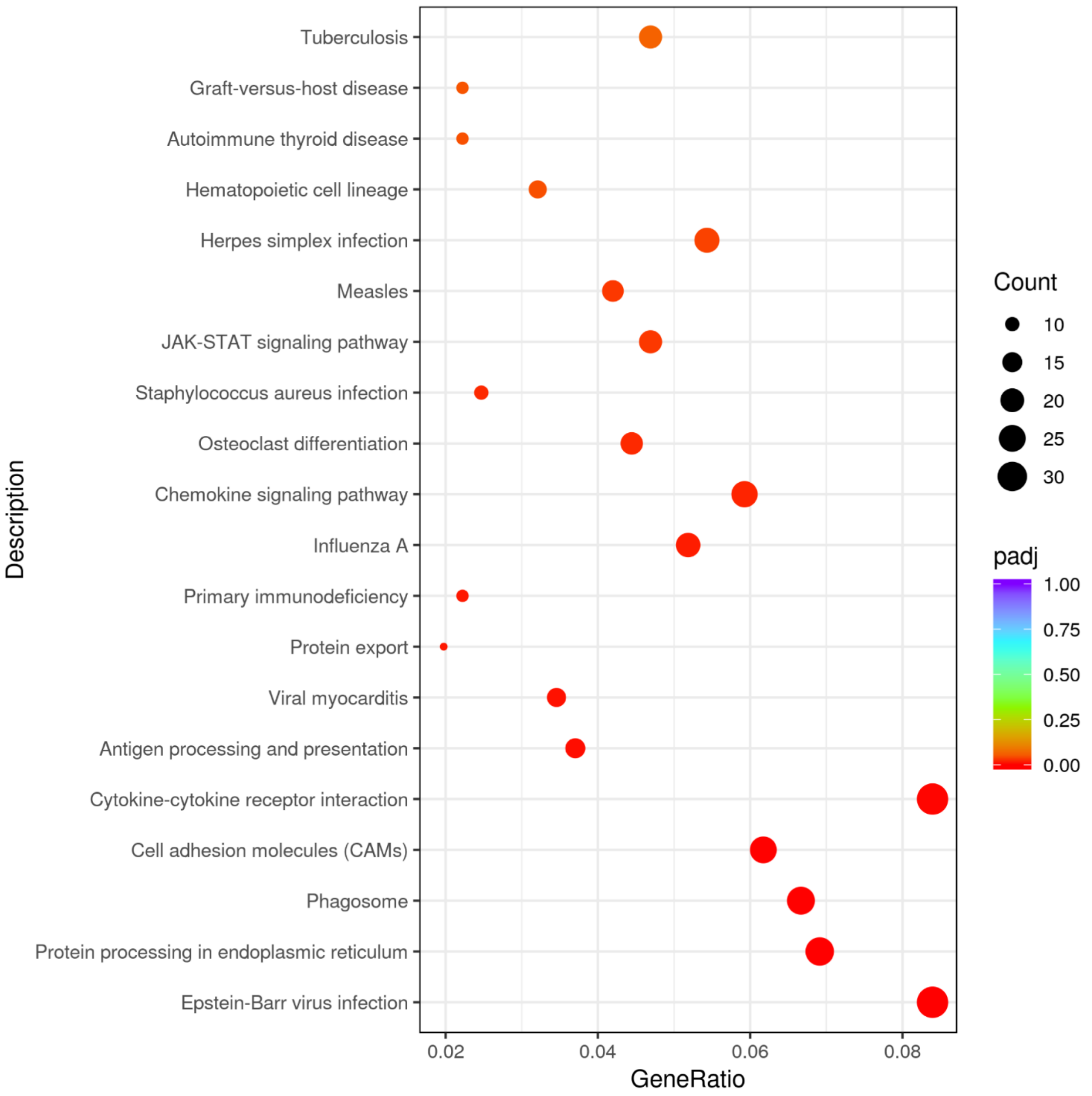

Supplemental Figure 5. KEGG pathway analysis of differentially expressed mRNAs between CTRL mice and CKO mice. Top 20 pathways were plotted according to enriched gene ratio and p value.
